# Differential Effects of Post-Weaning Diet and Maternal Obesity on Mouse Liver and Brain Metabolomes

**DOI:** 10.3390/nu12061572

**Published:** 2020-05-28

**Authors:** Sofiane Safi-Stibler, Etienne A. Thévenot, Luc Jouneau, Mélanie Jouin, Alexandre Seyer, Hélène Jammes, Delphine Rousseau-Ralliard, Christine Baly, Anne Gabory

**Affiliations:** 1 UVSQ, INRAE, BREED, Université Paris-Saclay, 78350 Jouy-en-Josas, France; sofiane.safi.stibler@gmail.com (S.S.-S.); luc.jouneau@inrae.fr (L.J.); melanie.jouin@inrae.fr (M.J.); helene.jammes@inrae.fr (H.J.); delphine.rousseau@inrae.fr (D.R.-R.); 2 Ecole Nationale Vétérinaire d’Alfort, BREED, 94700 Maisons-Alfort, France; 3 Collège Doctoral, Sorbonne Université, 75005 Paris, France; 4 CEA, LIST, Laboratory for Data Sciences and Decision, MetaboHUB, 91191 Gif-Sur-Yvette, France; Etienne.Thevenot@Cea.Fr; 5 Profilomic SA, 92100 Boulogne-Billancourt, 75008 Paris, France; alexandre.seyer@medday-pharma.com; 6 MedDay Pharmaceuticals, 75008 Paris, France; 7 NBO, INRAE, Université Paris-Saclay, 78350 Jouy-en-Josas, France

**Keywords:** high-fat diet, obesity, weight loss, liver, DOHaD, metabolomics

## Abstract

Nutritional changes during developmental windows are of particular concern in offspring metabolic disease. Questions are emerging concerning the role of maternal weight changes before conception, particularly for weight loss, in the development of diet-related disorders. Understanding the physiological pathways affected by the maternal trajectories in the offspring is therefore essential, but a broad overview is still lacking. We recently reported both metabolic and behavioral negative outcomes in offspring born to obese or weight-loss mothers and fed a control of high-fat diet, suggesting long-term modeling of metabolic pathways needing to be further characterized. Using non-targeted LC–HRMS, we investigated the impact of maternal and post-weaning metabolic status on the adult male offspring’s metabolome in three tissues involved in energy homeostasis: liver, hypothalamus and olfactory bulb. We showed that post-weaning diet interfered with the abundance of several metabolites, including 1,5-anhydroglucitol, saccharopine and β-hydroxybutyrate, differential in the three tissues. Moreover, maternal diet had a unique impact on the abundance of two metabolites in the liver. Particularly, anserine abundance, lowered by maternal obesity, was normalized by a preconceptional weight loss, whatever the post-weaning diet. This study is the first to identify a programming long-term effect of maternal preconception obesity on the offspring metabolome.

## 1. Introduction

In 2016, the worldwide prevalence of obesity in adult women was 15% [1]. Maternal obesity is a risk factor for metabolic and obstetric complications during pregnancy [2], but also for fetal outcomes because it is associated with a higher risk of the fetus being large or small for gestational age [3,4,5]. This affects fetal organogenesis and, thus, organ function after birth [6] and seems largely associated with the offspring’s long-term obesity risk, as stated by the concept of the developmental origins of health and disease (DOHaD) [7].

Given the many risks to both mother and child, overweight women are advised to lose weight before conception [8], although little is known about the consequences of a nutritional intervention, for fetal and tissue development or their long-term effects [9]. Fetal tissues adaption to changes in energy intake during key periods of gametogenesis and development can affect the functioning of key organs regulating energy metabolism and eating behavior, with long-term consequences [6,10]. However, few data are still available on the metabolomics consequences of maternal trajectories in adults. The liver (LI) controls metabolic homeostasis. Its morphology and functions are altered in utero in the context of maternal obesity and/or a high-fat diet (HFD), which contributes to the development of non-alcoholic fatty liver disease (NAFLD) in adulthood [11,12,13]. A maternal weight loss only partly normalizes the effects of obesity on fatty acid metabolism in lamb [14]. The hypothalamus (HYP) is a brain structure involved in the control of energy homeostasis and eating behavior. Its morphology and functions are also altered by obesity and/or maternal HFD [15,16], which can modify dietary and hedonic behaviors in adulthood [17,18,19]. Finally, the function of the olfactory system, which participates in the control of food intake, can also be altered by maternal HFD [20,21]. However, the consequences of a preconceptional maternal weight loss on brain metabolism and associated functions of the progeny remain poorly investigated [22,23].

We recently developed a mouse model of preconception maternal obesity and weight loss and characterized several metabolic and behavioral consequences at various ages in the offspring. In the short term, maternal obesity led to small-for-gestational-age fetuses, an effect reversed by preconception maternal weight loss [24]. In the long term, periconceptional maternal obesity was associated with a worsening of diet-induced obesity in male offspring challenged with an HFD from weaning to adulthood. Preconceptional weight loss normalized this phenotype, by improving male weight, but was associated with a modification of olfactory performances in the offspring, whatever the post-weaning diet: WL male offspring exhibited a lower peripheral olfactory sensitivity, and motivation to retrieve a hedonic food in WL mice under CD was not enhanced by fasting [21].

Here, we investigated the pathways underlying these metabolic and behavioral outcomes by using a non-targeted metabolomics approach, which can be useful in the field of DOHaD, to identify metabolic pathways in the offspring that are associated with maternal metabolism [25]. We thus used LC–HRMS to analyze the metabolome of three target tissues involved in food intake and nutrient management, LI, HYP and whole olfactory bulb (WOB) in adult male mice, according to maternal and post-weaning nutritional status. We noticed a large metabolic plasticity of the LI to high-fat diet as compared to limited changes in brain tissues. However, amino acid metabolism appeared as a key pathway mainly impacted by the high-fat diet in all tissues. Three discriminant metabolites were associated with the diet-induced obesity and correlated in the three tissues: 1,5-anhydroglucitol, saccharopine and β-hydroxybutyrate, highlighting the possible connections and dialog between tissues through blood. Among the two metabolites affected by the maternal diet in the LI, anserine abundancy appeared as a key marker of maternal obesity in the liver, whatever the post-weaning diet, and was normalized by weight loss, thus highlighting its putative role as a long-lasting programming factor of the adult hepatic phenotype. Altogether, our work adds new insights into metabolome changes in tissues rarely studied in the context of both maternal and offspring’s weight trajectories in a translational perspective connected with nutritional counselling in women of child-bearing age.

## 2. Materials and Methods 

### 2.1. Sample Processing and Preparation

**Figure 1:**
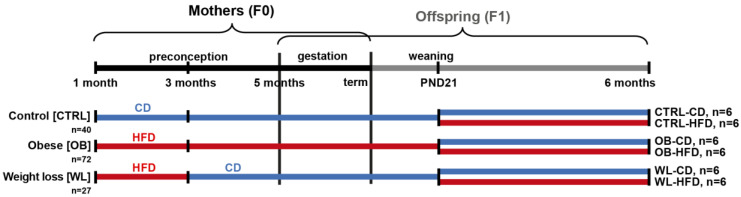
Animal model of preconception maternal weight trajectories. C57BL/6J F0 females were fed a control diet (CD, blue), a high-fat diet (HFD, red) or an HFD for two months and then switched onto the CD. On postnatal day 21 (PND21), half of the litter was weaned onto the HFD and the other half under the paired-CD diet, resulting in six groups: CD-CD, CD-HFD, OB-CD, OB-HFD, WL-CD and WL-HFD. All male offspring were followed until the age of six months. The number of animals studied (n) is indicated for each group.

The animal model, which received the approval (visa 12/062) of the COMETHEA ethical committee (Comité d’éthique pour l’expérimentation animale), registered with the Comité National de Réflexion Ethique sur l’Expérimentation Animale under the n°45, in accordance with European Union (Directive 2010/63/EU, 22 September 2010) legislation and the National Charter on the Ethics of Animal Experimentation, has been described elsewhere [21,24]. Briefly, as described in Figure 1, half of the C57BL/6J offspring mice born to control (CTRL), obese (OB) or weight-loss (WL) mothers were fed a control (CD: 20% kcal from proteins, 70% from carbohydrates and 10% from fat; #D12450K, Research Diets, New Brunswick, NJ, USA), and half were fed a high-fat diet (HFD: 20% proteins, 20% carbohydrates and 60% fat; #D12492). Full details of diet composition are described in [21]. 

Here, CD- or HFD-fed F1 males only were considered, as their adult phenotype was related to maternal conditioning [21]. From the whole cohort published in [21], six individuals of each group were selected by principal component analysis, as the closest to the barycenter of their own group (Supplementary Figure S1), after having excluded those presenting macroscopically apparent lesions at the time of death (malformation or nodules, which concerns up to 10% of C57BL/6 mice, as described in Harlan data sheet). These 6 animals were yet representative of their group, as they present statistical differences, like the whole initial population for all metabolic parameters previously investigated (Supplementary Table S1).

The mass spectrometry (MS) analysis was carried out by Profilomic (Saclay, France). The LI samples were ground in liquid N2, with a mortar and pestle. Ground LI, intact HYP and WOB tissues (~15 mg) were homogenized in ultrapure cold water with a sonicator (Vibra-Cell™ 75185, Sonic & Materials, Inc., Newton, 06470 CT, USA) and extracted by adding 600 µL of a cold methanol solution of an internal standard mixture (alanine ^13^C; metformin; ethylmalonic acid; aspartate ^15^N; glucose ^13^C; 2-aminoanthracene; amiloride; imipramine; atropine; ampicillin; prednisone; colchicine; dihydrostreptomycin; ATP ^15^N), incubated on ice for 90 min and centrifuged 10 min at 20,000 *g* at 4 °C. The aqueous phase was collected and evaporated to dryness under a stream of N2 (TurboVap^®^ LV, Biotage, Uppsala, Sweden). The dried samples were resuspended in 10 mM ammonium carbonate pH 10.5 and acetonitrile (40:60, *v*:*v*) and stored at −80 °C, until MS analysis.

### 2.2. Liquid Chromatography–High-Resolution Mass Spectrometry (LC–HRMS) Metabolite Analyses

LC–HRMS analysis and data processing were performed with a Q-Exactive™ Hybrid Quadrupole-Orbitrap™ mass spectrometer (Thermo Fisher Scientific, Les Ulis, France) coupled to a Transcend 1250 liquid chromatography system (Thermo Fisher Scientific, Les Ulis, France) equipped with an aSequant ZIC-pHILIC column (Merck). Samples were analyzed at high resolution (70,000 FWHM), with alternating positive and negative ionization modes. Raw data were processed with the XCMS software [26], and metabolites were annotated according to the exact mass and retention time in the Profilomic metabolite database. Annotated metabolites were compared with both the Human Metabolome Database (HMDB) [27] and the Chemical Entities of Biological Interest (ChEBI) database [28], to obtain accession numbers. MS/MS validation of metabolite annotations was performed by Profilomic on a subset of metabolites.

### 2.3. Biostatistical Analysis

Datasets for each tissue were analyzed separately. Statistical analyses were performed on the Workflow4Metabolomics online platform (W4M) [29,30]. Intensities were log10-transformed. Quality control was performed by using the Hotelling’s T2 test on the first plane of principal component analysis and the z-score of intensity deciles (threshold *p*-value = 0.001): one WL-CD sample and one CTRL-HFD sample were discarded from the LI dataset, one WL-CD sample from the HYP dataset and one WL-HFD sample from the WOB dataset. Partial least squares–discriminant analysis (PLS–DA) was performed for each tissue, with maternal group or post-weaning diet as a Y response. Transformed signals were mean-centered and divided by the standard deviation of each variable. For all validated models, the response variance explained (R2Y) and the predictive performance of the model (Q2Y) were shown significant by comparing the model built with the true response value and 10^3^ models built with random permutations of the response values [31]. Two-way analysis of variance (ANOVA) with interaction testing was performed for maternal group and post-weaning diet. We minimized the bias caused by the redundancy of metabolites detected in both ionization modes, by performing these analyses separately on metabolites detected in positive and negative ionization conditions. The *p*-values were adjusted for the false discovery rate (FDR) by the Benjamini–Hochberg procedure [32] and considered significant if ≤0.05. For metabolites displaying a maternal group effect or an interaction between maternal group and post-weaning diet, a post hoc test was performed on the raw signal intensity from MS peaks, with R software (version 3.4.3), using the lsmeans package (version 2.27-62) and adjustment by the Tukey method [33]. Correlation analyses were performed with the cor.test function, using the Pearson’s product-moment correlation method. When significant (*p* < 0.05), the correlation was considered very strong if |r| was greater than 0.65, strong if |r| was greater than 0.5, moderate if |r| was between 0.3 and 0.5 and weak for |r| values below 0.3.

### 2.4. Over-Representation Analysis

Datasets from each tissue were analyzed separately, using MetaboAnalyst [34]. The metabolite input type was HMDB accession numbers. Metabolites Set Enrichment Analysis/Over-Representation Analysis (MSEA–ORA) [35] was performed for the whole set of metabolites detected in each tissue, with the Profilomic metabolite database (960 compounds) as reference.

### 2.5. Pathway Analyses

Datasets from each tissue were analyzed separately, using MetaboAnalyst with HMDB accession numbers. The pathway library was the *Mus musculus* Kyoto Encyclopedia of Genes and Genomes (KEGG) [36] database. The selected pathway enrichment analysis method was “Global ANCOVA”, and the pathway topology analysis was “relative betweenness centrality”. Raw *p*-values were adjusted for FDR. We chose to exclude the ADP metabolite from the LI pathway analysis because of its key role in multiple metabolic pathways and enzymatic reactions. We also excluded metabolites with multiple annotations and metabolites for which the MS/MS signal did not correspond to any spectrum from the database. For the pathway analysis, we used the KEGG database [36] and the Small Molecule Pathway Database (SMPDB) [37]. The pathway impact score is calculated as the cumulative percentage for matched metabolite nodes, based on the importance of each node within a pathway [34].

## 3. Results

### 3.1. Nervous and Non-Nervous Tissues Display Similar Metabolite Profiles

**Figure 2:**
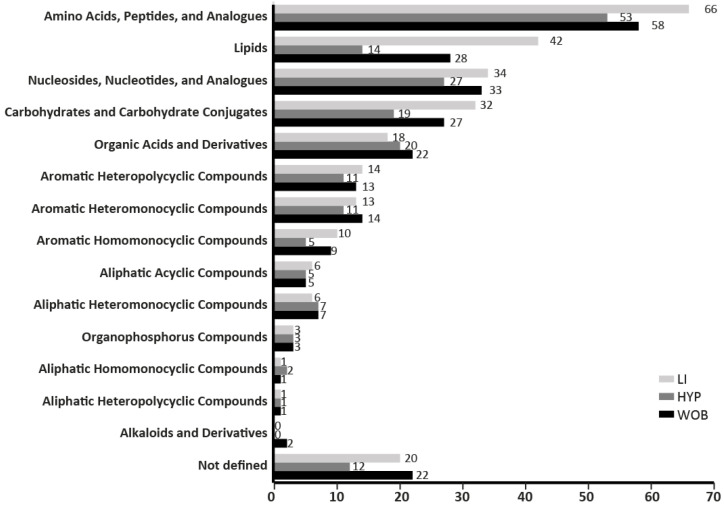
The number of metabolites categorized per chemical classes in the initial set of metabolites analyzed did not differ between the three tissues (liver LI, hypothalamus HYP and whole olfactory bulb WOB).

Metabolomics investigations were performed by LC–HRMS. Up to 471, 292 and 394 variables were annotated in positive and negative ionization mode, corresponding to 293, 206 and 271 unique metabolites, in the LI, HYP and WOB, respectively. Some of these had multiple annotations (between two and seven putative identities): 73 in the LI, 46 in the HYP and 68 in the WOB. Interestingly, the five main chemical metabolite classes were similar for all tissues: amino acids, carbohydrates, nucleosides, lipids and organic acids. The main difference between tissues was the number of metabolites in each chemical class (Figure 2).

### 3.2. Post-Weaning Diets Affect the Metabolite Profiles of both the Liver and Hypothalamus

**Figure 3:**
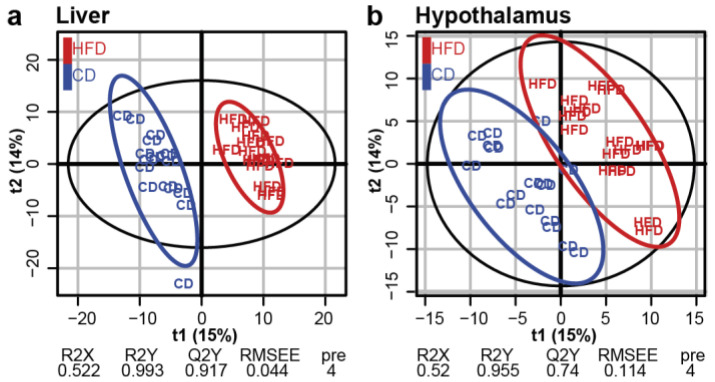
Post-weaning HFD had a global impact on the liver and hypothalamus metabolomes. Score plots from the PLS-DA classification into HFD and CD groups for the LI (**a**) and HYP (**b**). No model was built for WOB. A model is considered robust when the response variance explained (R2Y) is higher than the predictive performance of the model (Q2Y). A model with a Q2Y > 0.5 is considered to have a good predictive performance.

A PLS-DA was carried out to build, for each tissue, predictive models of maternal weight status or post-weaning diet. No model was significant in any tissue for the prediction of maternal group. In contrast, robust models for the prediction of post-weaning diet were obtained with the sets of metabolites detected in the LI and HYP (Figure 3a,b), but not in the WOB. The CD and HFD groups were separated on the first dimension (t1) for the LI (Q²Y = 0.917, *p* ≤ 0.001; Figure 3a) and on the two first dimensions (t1 and t2; Q²Y = 0.74, *p* ≤ 0.001; Figure 3b) for the HYP. In order to get a better view of the first dimension, an orthogonal PLS-DA (OPLS-DA) was carried out on each tissue (Supplementary Figure S2). In the LI and HYP, the OPLS-DA result was close to the PLS-DA, confirming the global impact of post-weaning diet. For the WOB, a model was built, in which robustness was not achieved, suggesting a global, but weaker effect of post-weaning diet in this tissue. To conclude, this descriptive analysis demonstrated a global effect of post-weaning diet on the metabolite profiles of the LI and the HYP.

As the effects of post-weaning diet may have hidden some subtle effects of maternal group, we carried out a more detailed evaluation of the effect of each condition and their interactions, with a differential analysis on each set of metabolites, based on two-way ANOVA.

### 3.3. Post-Weaning Diet Has a Major Impact on Metabolite Abundance in the Liver

**Figure 4:**
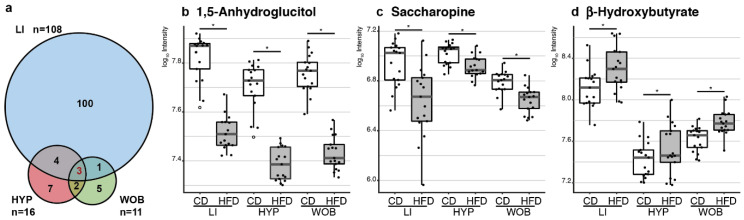
Post-weaning HFD discriminated several metabolites in the three tissues. (**a**) Venn diagram representing the number of metabolites discriminating between CD-fed and HFD-fed male mice. Abundance of the three metabolites affected similarly in the three tissues is represented as boxplot, with dots showing individual measurements, and white dots are outliers. (**b**) HFD-fed mice had significantly lower anhydroglucitol levels and (**c**) saccharopine levels than CD-fed mice. (**d**) HFD-fed mice had significantly higher β-hydroxybutyrate levels than CD-fed mice. * Indicates an adjusted *p*-value < 0.05.

In the LI, the metabolic profile differed considerably between post-weaning diets: Significant differences were observed for 108 metabolites, corresponding to more than one-third of the initial set (Figure 4a). The fold-change in levels ranged from 2.32 to −2.27, with four metabolites having absolute fold-change values >1.5. The abundance of 53 metabolites decreased in animals fed an HFD, whereas the abundance of 55 metabolites increased (Table 1). In the HYP and the WOB, the levels of 16 and 11 metabolites, respectively, were significantly affected by post-weaning diet, accounting for 8% and 4% of the initial set of metabolites, respectively (Figure 4a). The absolute fold-change values obtained were less than 1.5. Upregulation was observed for 13 out of 16 metabolites in the HYP and 4 out of 11 metabolites in the WOB (Table 1). The post-weaning diet therefore had a significant effect on the abundance of metabolites in the LI and, to a lesser extent, in the HYP and WOB.

The MSEA–ORA analysis showed no significant enrichment in any of the tissues studied (data not shown). Pathway analyses were not, therefore, biased by an over-representation of metabolites from a specific class. Functional pathway enrichment analysis, according to the KEGG database, revealed a major impact of post-weaning diet (impact score > 40%) in the LI on the metabolism of glutamine and glutamate, the biosynthesis of ubiquinone and other terpenoids-quinone, the metabolism of taurine and hypotaurine, β-alanine and methane. The metabolism of amino acids, lipids, carbohydrates and vitamin B was also altered, but to a lesser extent (impact score 10% to 40%; Table 2). In the HYP, according to the KEGG database, arginine and proline metabolism, the urea cycle and aspartate metabolism were significantly affected by the post-weaning diet (Table 3). Lysine degradation, pantothenate and CoA biosynthesis, and histidine metabolism were also affected by the post-weaning diet, but to a lesser extent. In the WOB, arginine and proline metabolism, pantothenate and CoA biosynthesis and lysine degradation were significantly affected by the post-weaning diet (Table 3). With the SMPDB database, the same pathways were retrieved in the HYP, and another affected pathway emerged in the WOB: alpha linolenic acid and linoleic acid metabolism (Table 3).

Three metabolites were affected by the post-weaning diet in all three tissues (Figure 4a): 1,5-anhydroglucitol (1,5-AG), saccharopine (Sacc) and β-hydroxybutyrate (β-HB). The abundance of 1,5-AG and Sacc was significantly lower in mice fed an HFD, whereas β-HB levels were higher, and the magnitude of this effect was similar in all three tissues (Figure 4b–d). Analyses of the correlation between the levels of each of these three metabolites in all tissues showed that the abundance of 1,5-AG was strongly and positively correlated between the three tissues, whereas the correlation of abundance between tissues was modest or weak for Sacc and β-HB (Supplementary Figure S3). Furthermore, analyses of the correlation between LI 1,5-AG and metabolic parameters, such as glycemia, insulinemia and area-under-curve for glycemia measured during an oral glucose tolerance test (OGTT), showed weak negative correlations in individuals fed a CD. In contrast, no correlation was observed in individuals under HFD (Supplementary Figure S4).

### 3.4. Maternal Diet Affects the Liver Abundance of Two Metabolites

**Figure 5:**
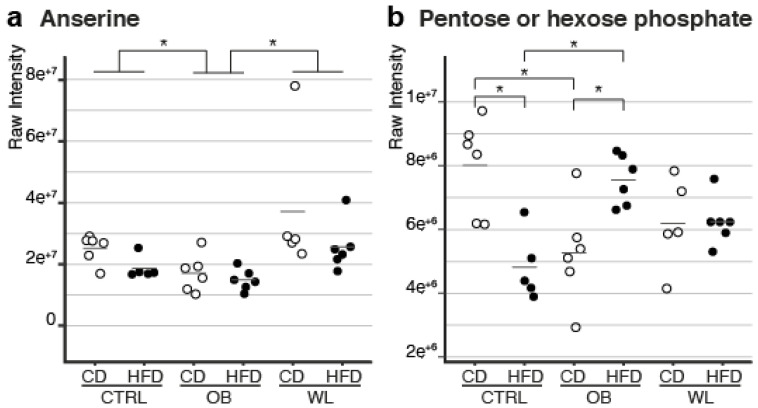
Two liver metabolite levels were affected by maternal diet group. (**a**) In the OB offspring, the abundance of anserine was significantly lower than that in CTRL and WL offspring. Anserine abundance did not differ significantly between the CTRL and WL offspring. This effect was independent of the post-weaning diet. (**b**) In the CTRL offspring, the abundance of P/H-P was significantly lower in animals fed the HFD. In the OB offspring, the opposite trend was observed: the abundance of P/H-P was significantly increased by the HFD. Furthermore, CTRL-CD and OB-CD offspring differed significantly from each other, as did CTRL-HFD and OB-HFD offspring. Finally, WL-CD and WL-HFD offspring were not significantly different from other offspring groups. Each dot represents an individual, and * indicates an adjusted *p*-value < 0.05.

The ANOVA analyses revealed an effect of maternal group on the metabolite profile for LI, through a modification of the level of one metabolite: anserine. Maternal obesity led to a lower anserine abundance as compared to CTRL (–28%) and WL (–48%) groups, whatever the post-weaning diet. Interestingly, preconception weight loss normalized its level (Figure 5a). In addition, there was an interaction between maternal group and post-weaning diet for one metabolite, a pentose or hexose phosphate (P/H-P) potentially corresponding to D-fructose-1-phosphate, D-fructose-6-phosphate, D-mannose-6-phosphate, galactose-1-phosphate, glucose-1-phosphate or mannose-1-phosphate. This interaction was well-illustrated by the opposite trends in its abundance observed in CTRL and OB offspring and by the lack of effect of the HFD context in WL offspring (Figure 5b).

## 4. Discussion

Our study reveals that maternal and postnatal metabolic trajectories have different impacts on metabolism in the LI, HYP and WOB and highlights pathways unique or common to these tissues. It is one of the rare studies to have compared the metabolomes of non-nervous and nervous system tissues, including one protected by a partial blood–brain barrier, and the first to have investigated the continuum between the HYP and the WOB in terms of their modulation by metabolic imbalance. Nervous tissues metabolomes have been poorly explored in a DOHaD context, mostly in cases of maternal intra-uterine malnutrition [38,39] or under-restriction [22], only one was dedicated to the effect of maternal obesity, but focused on fatty acids profile [23], and none explored the WOB, despite its unique metabolic network architecture and its importance as a metabolic sensor controlling food intake [40].

### 4.1. The Liver, Hypothalamus and Olfactory Bulb Metabolomes Were Principally Affected by Chronic HFD

This comparative metabolomics analysis supports our recent phenotypic analyses showing that the post-weaning diet had a major impact, whereas the maternal effect was more subtle [21]. A first striking result of this study was the similar impact of an HFD on three metabolites in the LI, HYP and WOB. Note that 1,5-AG has a structure similar to that of glucose, is produced by the LI and secreted into the bloodstream [41,42]. It is associated with diabetes [43], decreased in the blood of streptozotocin-induced diabetic mice [42]. In our model, the low abundance of 1,5-AG may result from the post-weaning HFD, since HFD-fed males display a diabetes-like phenotype. This hypothesis is also supported by the correlation between the three tissues for 1,5-AG abundance and with glucose metabolism markers in HFD context in the LI. We propose that a hepatic defect in HFD-fed males may affect the production of 1,5-AG, decreasing its concentration in the blood delivered to other tissues, as observed here for HYP and WOB.

Sacc is involved in gluconeogenesis, through its role in the propionyl-CoA synthesis pathway, and the TCA cycle, through its role in the acetyl-CoA production pathway (see KEGG, SMPDB and HMDB databases). The most over-represented pathways associated with metabolome variation in the three tissues belong to amino acid metabolism. The decrease in Sacc levels observed here may reflect an increase in amino acid degradation, affecting acetyl-CoA production and the TCA cycle, or the production of ketone bodies, such as β-HB, which has been implicated in energy production during fasting (see KEGG, SMPDB and HMDB). Fatty acids are converted by the LI into ketone bodies, which are released into the blood, when they are needed to be used by the tissues for energy production [44]. In rats, a Western diet induces an increase in β-HB levels in the cortex, with a cumulative effect with time of exposure, consistent with our observations [45]. The increase in β-HB abundance in HFD-fed males could have influenced neuronal activity and participated to behavioral outcomes. Indeed, β-HB intracerebroventricular injection increases orexigenic peptide production and food intake in mice [46], whereas it increases the insulin and leptin signaling potential in the HYP of diabetic rats [47]. β-HB is associated with GABAergic and glutamatergic signaling [48,49]. Moreover, the WOB expresses monocarboxylate and glucose transporters, which are sensitive to obesity context [50]. Therefore, chronic HFD may alter the sensing of energy needs, and an increase in β-HB, which can be seen as a signal of energy deprivation [51,52], would fuel a vicious cycle of food intake and disturbance associated with a decrease in olfactory performance observed in the whole population [21].

Apart from these three metabolites, each metabolome was affected by the post-weaning HFD in a different way. The LI metabolome displayed massive changes, whereas the HYP and WOB metabolomes were less affected. The LI metabolome is known to be more sensitive to HFD than those of the muscle or adipose tissues [53], highlighting its considerable plasticity in response to environmental challenges, which may reflect its key role in the control of homeostasis. HFD impacted metabolic pathways related to three main groups of compounds in the LI, amino acids, carbohydrates and lipids, and this finding is consistent with other findings in rodents. For example, HFD-fed male mice displaying negative endocrine hallmarks of obesity and hepatic steatosis also show a deregulation of the carbohydrates, lipids and amino acids metabolism [54]. A comparative study of steatohepatitis in rats and humans also reported a disturbance in amino acids, lipids and bile acids pathways [55]. Finally, since the plasma metabolomes of obese humans often display modifications in metabolites associated with lipids, carbohydrates, bile acids and amino acids [56], similar hepatic alterations among different species may have the same metabolic consequences, and the plasma metabolome reflects hepatic damages [57].

Hepatic metabolism is essential to supply the brain with energy and in signaling metabolites. Thus, HFD-induced hepatic damage could negatively affect brain metabolism and/or functions in the long term [58], even if, in juvenile mice fed a qualitatively different HFD for two months, HYP metabolome remains stable [59]. In our conditions, lysine degradation in HYP is of particular interest, as it is an essential amino acid for the brain, catabolized by the mitochondrial Sacc pathway. In adult neurons, Sacc could serve as a precursor for the de novo synthesis of glutamate, a major excitatory neurotransmitter in the brain [60,61]. Therefore, we speculate that HFD alters neuronal synaptic transmission in the HYP, acting through its dual impact on lysine degradation pathway and Sacc abundance. Alternatively, the alteration of the urea cycle and ammonia recycling could affect the production of ketone bodies from amino acids in the fasted state, helping the HYP to adjust its energetic needs, with an impact on the lysine pathway. Finally, the defect in ammonia recycling could illustrate the functional relationship between hepatic dysfunction and brain metabolites changes, as observed in cirrhotic animal models, as well as in human cerebrospinal fluid, in the context of hepatic encephalopathy [58,62].

The postnatal HFD also affected, to a lesser extent, the WOB metabolome, targeting the amino acid and fatty acid pathways. The activation of the mitochondrial Sacc pathway may have increased local glutamate production in the WOB, glutamate being involved in post-synaptic regulation of the olfactory message. We did not detect such a modification of glutamate production, but if it transiently occurred, it might have contributed to changes in olfactory perception, as observed in our model [21] and various others dealing with metabolic imbalances [40,50]. Alternatively, since amino acid- and fatty acid-sensing cells are abundant in the WOB [63] and HYP [64], any deregulation of their abundance would therefore modulate both neuronal and non-neuronal cell signaling pathways, adjusting feeding behavior according to metabolic needs, thereby lowering olfactory sensitivity. The increase in γ-linolenic acid levels and decreases in dihomo-γ-linolenic acid (8,11,14-eicosatrienoic acid) and eicosapentaenoic acid levels suggest that the linolenic acid pathway is modified in the WOB of HFD-fed animals (SMPDB database). These variations may affect the production of DHA, an essential long-chain polyunsaturated fatty acid (PUFA) of the n-3 series, involved in both membrane fluidity and neuroprotective/inflammatory processes in the brain [65,66]. This is consistent with results showing that obesity alters the serum long-chain PUFA profile [67], that moderate gestational dietary restriction alters their levels in the HYP, hippocampus and WOB of newborns [68] and that n-3 PUFA deficiency is associated with a decrease in olfactory performance [69].

### 4.2. The Maternal Environment Has a Persistent Effect on Metabolites in the Liver

Another originality of our study was to observe long-term effects of the maternal environment by a holistic metabolomic approach in offspring’s tissues. Only a few metabolomics studies have been performed in the context of DOHaD [70]. In the context of maternal obesity, most have focused on the placenta or biological fluids, such as serum [71,72]. One holistic study showed a programming effect of maternal obesity with gestational diabetes mellitus on the LI metabolome, but this metabolome was focused on the lipid extract [73]. To our knowledge, the only article reporting improvement of hepatic intracellular metabolism induced by maternal obesity upon maternal weight loss was performed in a non-human primate model at a fetal stage [74].

Outstandingly, we observed that the abundance of two metabolites in the LI was differential between maternal groups: anserine and a pentose or hexose phosphate; the latter could not be identified by MS/MS, and is therefore not discussed here. Anserine is the substrate of carnosinases, which convert it into methylhistidine and β-alanine [75,76]. Since the intestinal absorption of anserine is very stable [75], the variation reported here must be due to changes in its conversion. The low abundance of anserine may result from an increase in the activity of the conversion enzymes, which is consistent with the lower levels of methylhistidine in OB offspring. Alternatively, it may be due to a lower availability of substrates essentials for anserine production: carnosine and the methyl-donor *S*-adenosyl-methionine (SAM) (see KEGG and SMPDB databases). SAM levels did not differ between groups in our model, but the level of its product, *S*-adenosyl-homocysteine (SAH), was higher in HFD-fed offspring. SAH may also originate from the lysine-to-trimethyl lysine reaction of the carnitine synthesis pathway (see KEGG and SMPDB). Interestingly, trimethyl-lysine, carnitine and propionylcarnitine levels were all high in HFD-fed mice. Competition between the anserine and carnitine production pathways for SAM is consistent with the lower abundance of anserine in OB offspring. In various animal models, the induced hepatic steatosis was mitigated by histidine, carnosine or chicken liver (rich in anserine and carnosine) supplementation [77,78,79,80]. Anserine may therefore be considered as hepatoprotective. In our model, anserine levels were decreased in both CD- and HFD-fed OB males, but an increase in bodyweight was only observed for OB-HFD (Supplementary Table S1 and [21]). The low levels of anserine in OB offspring may have few metabolic consequences in a CD context, but they are consistent with the higher metabolic susceptibility of HFD-fed males. The LI of OB-HFD offspring may therefore be more rapidly affected by HFD-induced metabolic disorders, thus favoring NAFLD and the development of obesity. Anserine levels in WL offspring were similar to those in CTRL offspring, confirming our previous observation of the benefits of maternal weight loss for the offspring metabolic outcomes [21].

## 5. Conclusions

In conclusion, this study is the first to characterize the metabolome of three tissues in the same animal model, in the context of obesity. We identified several pathways, mainly linked to amino acid metabolism, in the three tissues tested, that support a major impact for post-weaning HFD on offspring’s trajectories. Our findings highlight the large metabolic plasticity of the LI and the relative homeostasis of the two central nervous system structures. Our study is also the first to report long-term effects of maternal preconception obesity on the adult offspring metabolome. Strikingly, anserine abundance was low in the LI of offspring born to obese mothers, potentially accounting for the greater diet-induced obesity conditioned by the maternal environment in our model. These findings open up new possibilities in the field of obesity management and in the research for the development of therapeutic approaches, to mitigate the detrimental effects of maternal obesity.

## Figures and Tables

**Table 1:** Discriminating metabolites between CD-fed and HFD-fed male mice.

	Decreased under HFD	Increased under HFD
Liver (LI)	**1,5-Anhydro-d-sorbitol; l-Saccharopine; Indoxyl-sulfate; Imidazolelactic-acid; Methylhippurate; 3-Methylhistidine; Methionine**; 2-Deoxyribose-5-phosphate; UMP; Inosine-5-monophosphate; GMP; 4-Guanidinobutyric-acid; 3-AMP/5-AMP; 4-Hydroxy-3-methoxyphenylglycol-sulfate; 3-Methylcrotonyl-glycine/*N*-Tiglylglycine; Guanine; l-Alanyl-l-proline; d-Glucosamine-6-phosphate; Glycerol-3-phosphate; Hydroxyphenylpyruvic-acid; Cytidine; ortho-Methylhippuric-acid/meta-Methylhippuric-acid/para-Methylhippuric-acid/Phenylacetylglycine; d-Fructose-1-phosphate/d-Fructose-6-phosphate/d-Mannose-6-phosphate/Galactose-1-phosphate/Glucose-1-phosphate/Glucose-6-PO4/Mannose-1-phosphate; Caprylolyglycine; UDP; Nervonic-acid; *N*-Acetyl-l-methionine; N-Isobutyrylglycine; 2-Oxobutyric-acid; *N*-Glycolylneuraminic-acid; 5-AMP/dGMP; Phosphonoacetic-acid; Guanosine-5-diphospho-d-mannose/Guanosine-5-diphosphoglucose; dGMP; Serine; Orotic-acid; Hexanoyl-glycine/*N*-Acetyl-d-allo-isoleucine/*N*-Acetyl-l-leucine; *N*-Acetyl-d-penicillamine/*N*-Acetyl-l-Methionine; Hexanoyl-glycine; Phosphoserine; Deoxyinosine; Uridine; Sebacic-acid; Guanosine-5-diphospho-l-fucose; 5-Aminolevulinic-acid/cis-4-hydroxy-d-proline/trans-3-*H*ydroxy-l-proline/trans-4-hydroxy-l-proline; Purine; Myristic-acid; *N*-Acetylneuraminic-acid; d-Pyroglutamic-acid; *Muramic-acid; Leu-Pro; Pyrrole-2-carboxylic-acid; D-Arabinose*	**3-Hydroxybutyric-acid; *N*-*N*-Dimethylglycine; Betaine; NAD; Taurine; 13-*S*-Hydroxyoctadeca-9Z-11E-dienoic-acid; Argininosuccinic-acid; 1-Methyladenosine; *S*-Adenosyl-homocysteine; Cyclic-ADP-ribose; dl-alpha-Hydroxystearic-acid; gamma-Linolenic-acid; β-Alanine; d-Threitol; Asparagine**; 3-Ureidopropionic-acid; Quinolinic-acid; l-Glutamic-acid; *N*-Methyl-d-aspartic-acid; Stachydrine; l-Cysteinesulfinic-acid; l-Kynurenine; Propionylcarnitine; ADP; 2-*O*-Methylinosine; Riboflavin; Pyridoxamine-5-phosphate; Sphinganine; 3-Hydroxy-2-methyl-butanoic-acid/3-Hydroxypentanoic-acid; Perillic-acid; l-Cysteic-acid; d-Sphingosine; 2-Aminopyridine-3-carboxylic-acid; Phosphoenolpyruvic-acid; 2-*O*-Methylguanosine; Fumaric-acid/Maleic-acid; 3-Hydroxypicolinic-acid; Uracil; Xanthosine; Diglycolic-acid/Malic-acid; d-Mannitol-1-phosphate; Prostaglandin-A1; Carnitine; Prostaglandin-E1; Indolelactic-acid; 5-Aminoimidazole-4-carboxamide-1b-d-ribofuranoside; Nicotinic-acid; 2-Hydroxyhexadecanoic-acid; Xanthine; N6-N6-N6-Trimethyl-l-lysine; Arachidic-acid; Cytidine-5-diphosphocholine; 4-Pyridoxic-acid; d-Glyceric-acid; *Aldosterone*
Hypothalamus (HYP)	**1,5-Anhydro-d-sorbitol** **; l-Saccharopine; 3-Methylhistidine**	**3-Hydroxybutyric-acid; Argininosuccinic-acid; Pantothenic-acid; Tyrosine; dl-Tryptophan; Phenylalanine; Arginine; Tartaric-acid; Valine; Asparagine**; Gly-Pro/Pro-Gly; *3-Aminosalicylic-acid; Leu-Pro*
Whole olfactory bulb (WOB)	**1,5-Anhydro-d-sorbitol; l-Saccharopine; cis-5,8,11,14,17-Eicosapentaenoic-acid; cis-8,11,14-Eicosatrienoic-acid; Lysine**; trans-4-hydroxy-l-proline; l-Homoserine/Threonine	**3-Hydroxybutyric-acid; gamma-Linolenic-acid; Pantothenic-acid**; *3-Aminosalicylic-acid*

When a compound has several putative annotations, they are separated by a “/”. Bold characters indicate metabolites validated by MS/MS; italic characters indicate metabolites that were not validated by MS/MS; regular characters indicate metabolites for which annotation could not be specified by MS/MS or was not tested. Underlined metabolites were affected similarly by the HFD in all three tissues.

**Table 2:** Pathway (KEGG) enrichment analysis in the liver: comparison between males fed an HFD and males fed a CD.

Pathways	Total Compounds	Hits	FDR	Impact	Metabolites
D-Glutamine and D-glutamate metabolism	5	1	0.00002	100%	L-Glutamic acid ↑
Ubiquinone and other terpenoid-quinone biosynthesis	3	1	0.00644	100%	4-Hydroxyphenylpyruvic acid ↓
Taurine and hypotaurine metabolism	8	3	0.00002	71%	Cysteic acid ↑; 3-Sulfinoalanine ↑; Taurine ↑
Beta-alanine metabolism	17	3	0.00001	67%	Beta-alanine ↑; Ureidopropionic acid ↑; Uracil ↑
Methane metabolism	9	1	0.02049	40%	L-Serine ↓
Glycine, serine and threonine metabolism	31	5	0.00001	36%	Dimethylglycine ↑; Phosphoserine ↓
Purine metabolism	68	8	0.00001	28%	Xanthine ↑; AICAR ↑; Inosinic acid ↓; Deoxyinosine ↓; Xanthosine ↑; Guanosine monophosphate ↓; Guanine ↓; 2′-Deoxyguanosine 5′-monophosphate ↓
Pyrimidine metabolism	41	8	0.00001	28%	Uridine 5′-diphosphate ↓; Uridine 5′-monophosphate ↓; Uridine ↑; Ureidopropionic acid ↑; Cytidine ↑; Orotic acid ↑; Uracil ↑; Beta-Alanine ↑
Alanine, aspartate and glutamate metabolism	24	4	0.00001	28%	Argininosuccinic acid ↑; L-Glutamic acid ↑; L-Asparagine ↓; Glucosamine 6-phosphate ↓
Nicotinate and nicotinamide metabolism	13	3	0.00001	21%	Quinolinic acid ↑; NAD ↑; Nicotinic acid ↑
Cysteine and methionine metabolism	27	6	0.00001	20%	L-Serine ↓; L-Methionine ↑; S-Adenosylhomocysteine ↑; Cysteic acid ↑; 3-Sulfinoalanine ↑; 2-Ketobutyric acid ↓
Sphingolipid metabolism	21	3	0.00005	20%	Sphinganine ↑; L-Serine ↓; Sphingosine ↑
Glycerolipid metabolism	18	2	0.00019	13%	Glycerol 3-phosphate ↓; Glyceric acid ↑
Glycerophospholipid metabolism	30	2	0.00012	13%	Citicoline ↑; Glycerol 3-phosphate ↓
Aminoacyl-tRNA biosynthesis	69	4	0.00038	13%	L-Asparagine ↓; L-Serine ↓; L-Methionine ↑; L-Glutamic acid ↑
Arginine and proline metabolism	44	3	0.00001	12%	Argininosuccinic acid ↑; L-Glutamic acid ↑; 4-Guanidinobutanoic acid ↓
Tryptophan metabolism	40	1	0.00056	11%	L-Kynurenine ↓
Glycolysis or gluconeogenesis	26	1	0.00032	10%	Phosphoenolpyruvic acid ↑
Amino sugar and nucleotide sugar metabolism	37	3	0.00020	8%	Glucosamine 6-phosphate ↓; GDP-L-fucose ↓; *N*-Glycolylneuraminic acid ↓
Tyrosine metabolism	44	1	0.00645	7%	4-Hydroxyphenylpyruvic acid ↓
Pentose phosphate pathway	19	1	0.00002	7%	Deoxyribose 5-phosphate ↓
Glyoxylate and dicarboxylate metabolism	18	1	0.00032	6%	Glyceric acid ↑
Glutathione metabolism	26	1	0.00002	6%	L-Glutamic acid ↑
Vitamin B6 metabolism	9	2	0.00002	5%	Pyridoxamine 5′-phosphate ↑; 4-Pyridoxic acid ↑
Pantothenate and CoA biosynthesis	15	3	0.00001	4%	Ureidopropionic acid ↑; Beta-Alanine ↑; Uracil ↑
Primary bile acid biosynthesis	46	1	0.00002	3%	Taurine ↑
Lysine degradation	23	2	0.00001	1%	N6,N6,N6-Trimethyl-L-lysine ↑; Saccharopine ↓
Histidine metabolism	15	2	0.00001	0%	L-Glutamic acid ↑; 1-Methylhistidine ↓
Biosynthesis of unsaturated fatty acids	42	3	0.00001	0%	Nervonic acid ↓; Arachidic acid ↑; Gamma-Linolenic acid ↑
Butanoate metabolism	22	1	0.00002	0%	L-Glutamic acid ↑
Porphyrin and chlorophyll metabolism	27	1	0.00002	0%	L-Glutamic acid ↑
Nitrogen metabolism	9	1	0.00002	0%	L-Glutamic acid ↑
Propanoate metabolism	20	2	0.00003	0%	Beta-Alanine ↑; 2-Ketobutyric acid ↓
Linoleic acid metabolism	6	1	0.00003	0%	13S-hydroxyoctadecadienoic acid ↑
Limonene and pinene degradation	8	1	0.00011	0%	Perillic acid ↑
Riboflavin metabolism	11	1	0.00012	0%	Riboflavin ↑
Citrate cycle (TCA cycle)	20	1	0.00032	0%	Phosphoenolpyruvic acid ↑
Pyruvate metabolism	23	1	0.00032	0%	Phosphoenolpyruvic acid ↑
Lysine biosynthesis	4	1	0.00466	0%	Saccharopine ↓
Phenylalanine, tyrosine and tryptophan biosynthesis	4	1	0.00645	0%	4-Hydroxyphenylpyruvic acid ↓
Cyanoamino acid metabolism	6	1	0.02049	0%	L-Serine ↓

**Table 3:** Pathway (KEGG and SMPDB) enrichment analysis in the hypothalamus and whole olfactory bulb: comparison between males fed an HFD and males fed a CD.

Pathways	Total Compounds	Hits	FDR	Impact	Metabolites
**Hypothalamus (KEGG and SMPDB databases retrieved the same results)**					
Aspartate metabolism	34	3	0.00818	35%	L-Asparagine ↑, Argininosuccinic acid ↑, L-Arginine ↑
Arginine and proline metabolism	48	2	0.00586	24%	Argininosuccinic acid ↑, L-Arginine ↑
Urea cycle	23	2	0.00586	22%	Argininosuccinic acid ↑, L-Arginine ↑
Phenylalanine and tyrosine metabolism	25	2	0.01921	12%	L-Phenylalanine ↑, L-Tyrosine ↑
Pantothenate and CoA biosynthesis	19	1	0.00586	7%	Pantothenic acid ↑
Ammonia recycling	25	1	0.03670	3%	L-Asparagine ↑
Lysine degradation	20	1	0.01921	3%	Saccharopine ↓
Beta-alanine metabolism	26	2	0.00586	0%	3-Methylhistidine ↑, Pantothenic acid ↑
Valine, leucine and isoleucine degradation	51	1	0.02030	0%	L-Valine ↑
Catecholamine biosynthesis	14	1	0.03065	0%	L-Tyrosine ↑
Tyrosine metabolism	55	1	0.03065	0%	L-Tyrosine ↑
**Whole olfactory bulb (KEGG database)**					
Arginine and proline metabolism	44	1	0.00233	4%	Hydroxyproline ↓
Pantothenate and CoA biosynthesis	15	1	0.00430	2%	Pantothenic acid ↑
Lysine degradation	23	2	0.00015	1%	L-Lysine ↓; Saccharopine ↓
Biosynthesis of unsaturated fatty acids	42	3	0.00015	0%	8,11,14-Eicosatrienoic acid ↓; Gamma-linolenic acid ↑; Eicosapentaenoic acid ↓
Lysine biosynthesis	4	2	0.00015	0%	L-Lysine ↓; Saccharopine ↓
Biotin metabolism	5	1	0.00430	0%	L-Lysine ↓
Aminoacyl-tRNA biosynthesis	69	1	0.00430	0%	L-Lysine ↓
**Whole olfactory bulb (SMPDB database)**					
Alpha linolenic acid and linoleic acid metabolism	17	3	0.00032	26%	Eicosapentaenoic acid ↓; 8,11,14-Eicosatrienoic acid ↓; Gamma-Linolenic acid ↑
Lysine degradation	20	2	0.18397	3%	L-Lysine ↓; Saccharopine ↓
Biotin metabolism	7	1	0.72255	0%	L-Lysine ↓
Carnitine synthesis	16	1	0.72255	0%	L-Lysine ↓

## Supplementary Materials

The following are available online at https://www.mdpi.com/2072-6643/12/6/1572/s1. Table S1: Phenotypic characteristics of the male mice for the whole population and the six selected individuals per group. Figure S1: Selection of the individuals for LC–HRMS. Figure S2: Score plots from the OPLS-DA classification, with one orthogonal component, into HFD and CD groups for the liver (a), hypothalamus (b) and whole olfactory bulb (c). Figure S3: Between-tissue correlation for 1,5-anhydroglucitol, saccharopine and β-hydroxybutyrate. Figure S4: Correlation between 1,5-anhydroglucitol signal and parameters of the glucose metabolism (fasting glycemia, fasting insulinemia and area-under-curve (AUC)) for glycemia measured during an OGTT. An Excel file containing LC–HRMS data is provided as “Safi-Stibler_lchrms_data.xlsx”. An Excel file containing fold change, coefficient of variation and *p*-values is provided as “Safi-Stibler_Statistical_Analysis_results.xlsx”.
