# Supplementary material for: Differential Effects of Post-Weaning Diet and Maternal Obesity on Mouse Liver and Brain Metabolomes
Source: Nutrients. 2020 May 28;12(6):1572. doi: 10.3390/nu12061572 (PMC7352523; doi:10.3390/nu12061572)

Supplementary table T1 : Phenotypic characteristics of the male mice for the whole population and for the 6 selected individuals per group.
The grey lines recall the statistical analyses for the whole group population, as described in Panchenko et al, Nutrients, 2019,
and the white lines give those for the 6 individuals per group, as selected with the method described in supplementary figure 1 below.
For all the metabolic parameters, the p-values on the selected individuals show the same level of significance than for the whole population (in red). For behavioral parameters, the significance between HFD and CD is not reached for the hidden cheese test for the selected individuals, although it follows the same tendency (in orange).
NS: non-significant.

	Metabolic parameters														Behavioural parameters			
	Weight (g)	WATSC/BW	WATPG/BW	WATPR/BW	TotalWAT/BW	KIDNEYS/BW	HEART/BW	BAT/BW	LIVER/BW	Cholesterolemia (mmol/L)	Fasting Glycemia (mg/dL)	AUC (mg.min/dL)	Fasting Insulinemia (pmol/L)	Leptinemia (ng/mL)	Food Intake (Kcal/kg BW/d)	Hidden Cheese Test (s)	Olfactory Preference	EOG amplitude (mV)
Mean CTRL-CD whole population (n=14)	28.58 ± 2.66	0.0253 ± 0.0082	0.0326 ± 0.0130	0.0135 ± 0.0056	0.0714 ± 0.0258	0.0117 ± 0.0020	0.0053 ± 0.0009	0.0051 ± 0.0015	0.0341 ± 0.0136	2.961 ± 1.474	155.21 ± 15.30	21897 ± 2941	158.11 ± 61.90	7.31 ± 5.67	437.32 ± 79.00	212.29 ± 90.15	0.530 ± 0.294	16.388 ± 3.612
Mean CTRL-CD individuals (n=6)	28.49 ± 2.94	0.0262 ± 0.0034	0.0355 ± 0.0117	0.0151 ± 0.0052	0.0767 ± 0.0194	0.0105 ± 0.0006	0.0048 ± 0.0004	0.0058 ± 0.0016	0.0391 ± 0.0035	4.021 ± 0.489	159.67 ± 11.90	23654 ± 761	155.75 ± 34.69	8.79 ± 6.44	405.75 ± 30.37	190.00 ± 107.29	0.599 ± 0.350	15.292 ± 2.111
Mean CTRL-HFD whole population (n=17)	41.71 ± 5.46	0.0675 ± 0.0094	0.0575 ± 0.0162	0.0334 ± 0.0062	0.1584 ± 0.0157	0.0086 ± 0.0011	0.0041 ± 0.0007	0.0070 ± 0.0027	0.0324 ± 0.0088	5.582 ± 1.486	192.53 ± 43.98	30781 ± 5424	632.89 ± 382.26	58.12 ± 19.31	325.21 ± 41.24	245.38 ± 88.60	0.68 ± 0.221	14.987 ± 1.818
Mean CTRL-HFD individuals (n=6)	41.13 ± 4.75	0.0635 ± 0.0091	0.0604 ± 0.0143	0.0326 ± 0.0041	0.1565 ± 0.0083	0.0086 ± 0.0007	0.0041 ± 0.0005	0.0069 ± 0.0022	0.0314 ± 0.0064	5.361 ± 1.456	175.90 ± 19.37	29920 ± 1608	596.13 ± 301.13	55.22 ± 21.87	331.05 ± 36.22	244.40 ± 79.84	0.562 ± 0.128	13.256 ± 0.659
Mean OB-CD whole population (n=9)	28.97 ± 2.79	0.0233 ± 0.0046	0.0310 ± 0.0109	0.0115 ± 0.0031	0.0658 ± 0.0172	0.0126 ± 0.0017	0.0059 ± 0.0010	0.0047 ± 0.0009	0.0347 ± 0.0069	3.005 ± 1.159	154.22 ± 17.77	21075 ± 2648	151.8 ± 38.98	5.97 ± 3.12	466.10 ± 79.21	202.89 ± 104.01	0.444 ± 0.266	14.234 ± 4.033
Mean OB-CD individuals (n=6)	28.40 ± 2.28	0.0238 ± 0.0052	0.0333 ± 0.0116	0.0117 ± 0.0030	0.0689 ± 0.0180	0.0124 ± 0.0014	0.0056 ± 0.0011	0.0048 ± 0.0009	0.0357 ± 0.0069	2.964 ± 1.222	155.42 ± 18.89	21541 ± 3014	163.69 ± 39.27	5.70 ± 3.24	452.24 ± 82.89	193.00 ± 106.02	0.419 ± 0.330	13.587 ± 4.498
Mean OB-HFD whole population (n=9)	46.34 ± 2.79	0.0751 ± 0.0141	0.0487 ± 0.0074	0.0402 ± 0.0069	0.1640 ± 0.0197	0.0091 ± 0.0011	0.0042 ± 0.0008	0.0087 ± 0.0047	0.0342 ± 0.0059	5.950 ± 1.842	198.83 ± 19.16	35913 ± 7126	667.91 ± 314.17	68.47 ± 15.31	311.72 ± 39.34	287.33 ± 38.00	0.611 ± 0.294	14.783 ± 2.530
Mean OB-HFD individuals (n=6)	47.37 ± 1.85	0.0721 ± 0.0113	0.0476 ± 0.0062	0.0427 ± 0.0056	0.1624 ± 0.0134	0.0087 ± 0.0011	0.0038 ± 0.0007	0.0105 ± 0.0048	0.0330 ± 0.0048	5.795 ± 2.206	198.25 ± 11.12	34724 ± 5184	786.72 ± 348.12	66.65 ± 18.05	313.23 ± 44.50	281.00 ± 46.54	0.561 ± 0.329	15.853 ± 0.945
Mean WL-CD whole population (n=17)	27.44 ± 3.40	0.0249 ± 0.0076	0.0294 ± 0.0097	0.0127 ± 0.0076	0.0680 ± 0.0237	0.0107 ± 0.0011	0.0051 ± 0.0005	0.0049 ± 0.0016	0.0350 ± 0.0043	3.114 ± 0.957	150.34 ± 23.43	21582 ± 3809	189.77 ± 80.06	7.79 ± 8.51	403.48 ± 49.23	149.63 ± 97.80	0.399 ± 0.395	14.445 ± 3.976
Mean WL-CD individuals (n=6)	27.21 ± 1.99	0.0233 ± 0.0045	0.0275 ± 0.0042	0.0115 ± 0.0010	0.0647 ± 0.0068	0.0108 ± 0.0004	0.0051 ± 0.0004	0.0045 ± 0.0008	0.0358 ± 0.0009	3.379 ± 0.562	149.50 ± 15.19	21465 ± 1585	154.32 ± 65.86	5.60 ± 2.89	412.31 ± 29.63	206.00 ± 106.62	0.469 ± 0.505	15.9841 ± 1.553
Mean WL-HFD whole population (n=19)	41.31 ± 3.92	0.0658 ± 0.0099	0.0632 ± 0.0135	0.0308 ± 0.0062	0.1598 ± 0.0150	0.0083 ± 0.0006	0.0037 ± 0.0003	0.005 ± 0.0024	0.0306 ± 0.0047	5.616 ± 1.337	185.89 ± 18.47	35228 ± 8740	522.70 ± 244.40	60.34 ± 23.86	326.89 ± 38.32	229.53 ± 101.53	0.423 ± 0.260	13.338 ± 4.243
Mean WL-HFD individuals (n=6)	41.91 ± 4.06	0.0666 ± 0.0097	0.0672 ± 0.0150	0.0335 ± 0.0040	0.1674 ± 0.0124	0.0082 ± 0.0007	0.0036 ± 0.0004	0.0052 ± 0.0020	0.0297 ± 0.0014	5.725 ± 0.775	184.08 ± 11.91	31750 ± 2995	467.16 ± 173.20	58.00 ± 18.76	333.01 ± 37.40	245.17 ± 102.08	0.335 ± 0.276	NA
pval **whole population maternal group (Panchenko et al, 2019)**	*0.031*	*NS*	*NS*	*NS*	*NS*	*0.028*	*0.041* *WL vs OB p=0.001*	*NS*	*NS*	*NS*	*NS*	*NS*	*NS*	*NS*	*NS*	*NS*	*NS*	*0.012*
pval **individuals maternal group**	*0.03825* *post-hoc NS*	*NS*	*NS*	*NS*	*NS*	*0.0101* *post-hoc* *OB vs CTRL p=0.0312* *WL vs OB p=0.0335*	*NS*	*0.0456* *post-hoc* *WL vs OB p=0.0355*	*NS*	*NS*	*NS*	*NS*	*NS*	*NS*	*NS*	*NS*	*NS*	*NS*
pval **whole population HFD vs CD (Panchenko et al, 2019)**	*<0.01*	*<0.01*	*<0.01*	*<0.01*	*<0.01*	*<0.01*	*<0.01*	*<0.01*	*<0.01*	*<0.01*	*<0.01*	*<0.01*	*<0.01*	*<0.01*	*<0.01*	*2.00E-16*	*NS*	*NS*
mean CD individuals (n=18)	28.03 ± 2.36	0.0244 ± 0.0043	0.0321 ± 0.0098	0.0128 ± 0.0038	0.0704 ± 0.0160	0.0113 ± 0.0013	0.0052 ± 0.0007	0.0050 ± 0.0012	0.0369 ± 0.0045	3.455 ± 0.896	154.86 ± 15.26	22264 ± 2183	157.58 ± 46.20	6.69 ± 4.48	423.43 ± 54.75	195.76 ± 100.00	0.497 ± 0.377	14.611 ± 3.304
mean HFD individuals (n=18)	43.47 ± 4.54	0.0674 ± 0.0101	0.0584 ± 0.0144	0.0363 ± 0.0064	0.1621 ± 0.0118	0.0085 ± 0.0008	0.0039 ± 0.0006	0.0076 ± 0.0039	0.0314 ± 0.0046	5.627 ± 1.506	186.68 ± 16.30	32261 ± 3991	615.38 ± 296.31	59.96 ± 19.11	325.76 ± 38.26	257.59 ± 76.45	0.561 ± 0.256	14.544 ± 1.842
pval **individuals HFD vs CD**	*5.748E-15*	*<2e-16*	*2.893E-07*	*8.365E-15*	*<2e-16*	*1.64E-09*	*7.574E-07*	*0.0075*	*9.60E-04*	*1.36E-05*	*1.20E-06*	*5.462E-10*	*6.164E-07*	*1.054E-12*	*8.834E-07*	*0.06044*	*NS*	*NS*

Supplementary figure S1: Selection of the individuals for LC-HRMS.

The Principal Component Analysis analyzing all the phenotypic characteristics listed in the supplementary table 1, allowed the selection of 6 males per group. Were selected the 6 individuals whose coordinates are closer to the barycenter of their own group (represented with an "x")

Each circle represents one individual, the color of which is according to its group in the legend. The plain circles represent the 6 selected individuals.

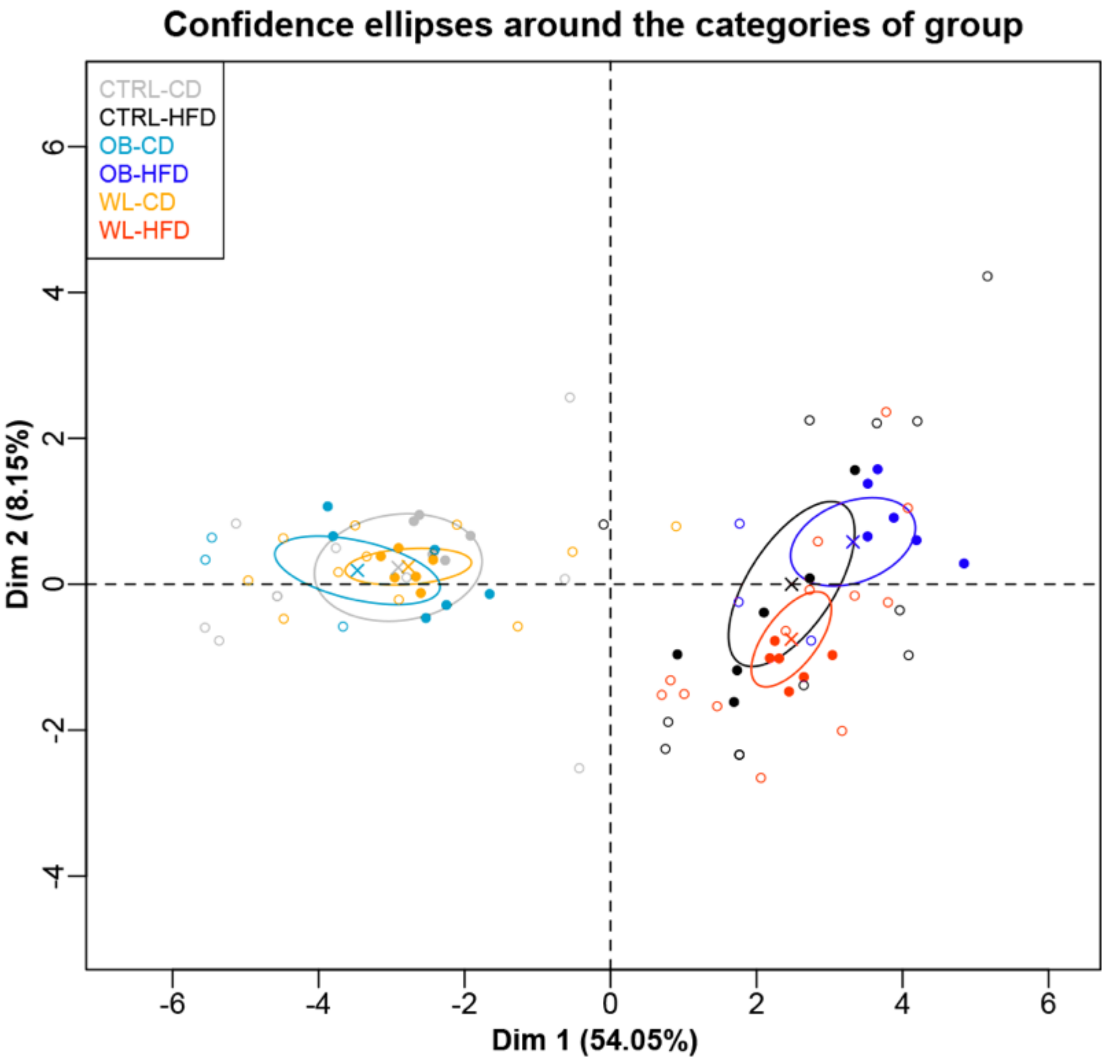

Supplementary Figure S2: Score plots from the OPLS-DA classification, with one orthogonal component, into HFD and CD groups for the liver (a), hypothalamus (b) and whole olfactory bulb (c). A model is considered robust when the response variance explained (R2Y) is higher than the predictive performance of the model (Q2Y). For the olfactory bulb, the model is robust but have a low predictive performance (Q2Y<0.5)

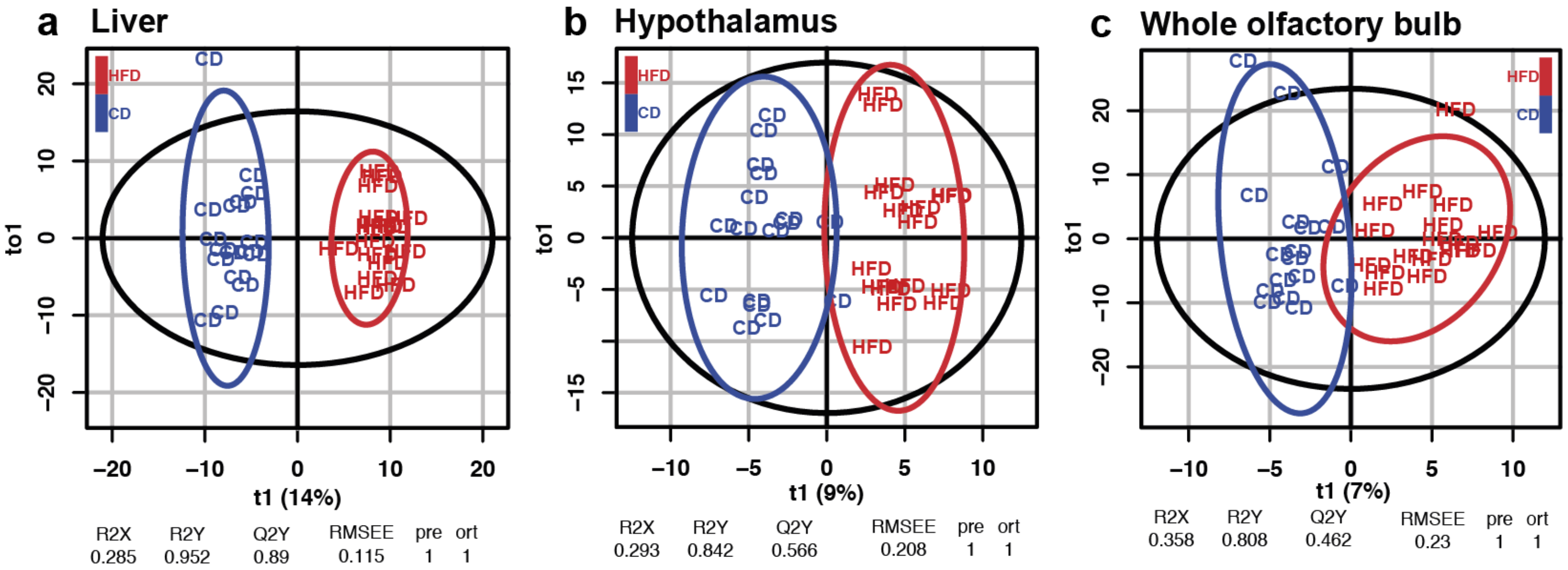

Supplementary Figure S3:
Between-tissue correlation for 1,5-Anhydroglucitol, Saccharopine and β-hydroxybutyrate

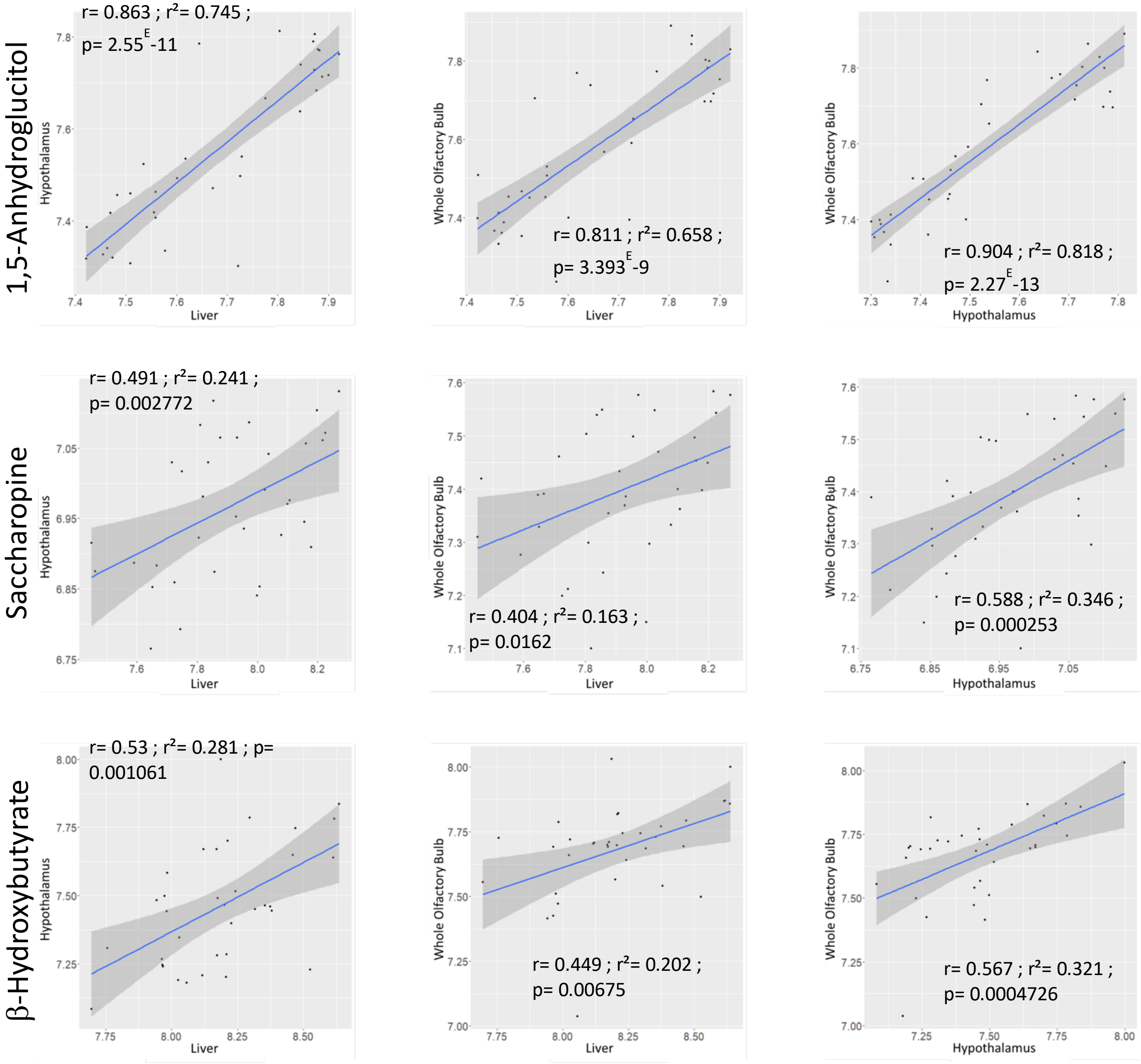

Supplementary figure S4: Correlation between 1,5-anhydroglucitol signal and parameters of the glucose metabolism (fasting glycemia, fasting insulinemia and area-under-curve (AUC) for glycemia measured during an OGTT.

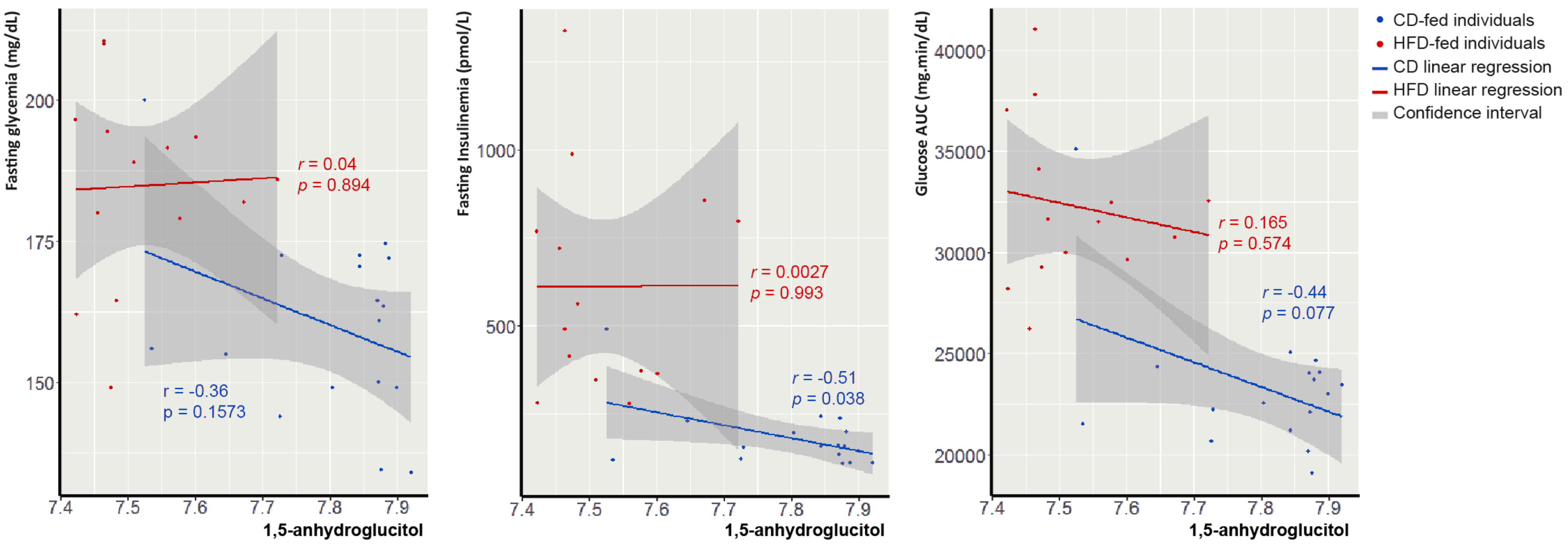

Supplement: Supplementary file 1 [file nutrients-12-01572-s001.zip › Safi-Stibler_Suppl Fig.pdf]
